# Autoimmunity Increases Susceptibility to and Mortality from Sepsis

**DOI:** 10.4049/immunohorizons.2100070

**Published:** 2021-10-26

**Authors:** Isaac J. Jensen, Samantha N. Jensen, Patrick W. McGonagill, Thomas S. Griffith, Ashutosh K. Mangalam, Vladimir P. Badovinac

**Affiliations:** * Interdisciplinary Graduate Program in Immunology, University of Iowa, Iowa City, IA; † Department of Pathology, University of Iowa, Iowa City, IA; ‡ Department of Surgery, University of Iowa, Iowa City, IA; § Microbiology, Immunology, and Cancer Biology Ph.D. Program, University of Minnesota, Minneapolis, MN; ¶ Department of Urology, University of Minnesota, Minneapolis, MN; ∥ Center for Immunology, University of Minnesota, Minneapolis, MN; # Masonic Cancer Center, University of Minnesota, Minneapolis, MN; ** Minneapolis Veterans Affairs Health Care System, Minneapolis, MN; †† Department of Microbiology and Immunology, University of Iowa, Iowa City, IA

## Abstract

We recently demonstrated how sepsis influences the subsequent development of experimental autoimmune encephalomyelitis (EAE) presented a conceptual advance in understanding the postsepsis chronic immunoparalysis state. However, the reverse scenario (autoimmunity prior to sepsis) defines a high-risk patient population whose susceptibility to sepsis remains poorly defined. In this study, we present a retrospective analysis of University of Iowa Hospital and Clinics patients demonstrating increased sepsis prevalence among multiple sclerosis (MS), relative to non-MS, patients. To interrogate how autoimmune disease influences host susceptibility to sepsis, well-established murine models of MS and sepsis and EAE and cecal ligation and puncture, respectively, were used. EAE, relative to non-EAE, mice were highly susceptible to sepsis-induced mortality with elevated cytokine storms. These results were further recapitulated in LPS and *Streptococcus pneumoniae* sepsis models. This work highlights both the relevance of identifying highly susceptible patient populations and expands the growing body of literature that host immune status at the time of septic insult is a potent mortality determinant.

## INTRODUCTION

Multiple sclerosis (MS) is an autoimmune demyelinating disease of the CNS that affects ~2.8 million individuals worldwide, and cases are rising (1, 2). The symptomology of MS includes (but is not limited to) pain, motor dysfunction, and cognitive dysfunction. The cause of MS is not well understood but is thought to stem from a complex interaction of genetic and environmental factors (3, 4). MS is commonly diagnosed between the ages of 20–40, although underlying subclinical pathogenesis may be present long before diagnosis. MS pathogenesis is mediated by proinflammatory autoreactive T cells and other immune cells activated prior to migration into the CNS to promote axonal damage (1). In an attempt to subvert the aberrant immune response to the CNS, immunomodulatory/immunosuppressive drugs are often prescribed to patients with MS with varying degrees of success (5). Unfortunately, the use of disease-modifying drugs in patients with MS often comes with an increased risk of opportunistic infection (6). The increased propensity to infection may leave MS patients at an increased risk of sepsis (7).

Sepsis, a dysregulated host response to infection, impacts nine people every 6 s, of which two will succumb to the associated cytokine storm (8). Additionally, those who survive demonstrate an increased susceptibility to subsequent infection or cancer development (9–12). This increased risk for secondary complication leads to a substantial economic burden costing over $20 billion annually in the United States alone (13). Although mortality due to the cytokine storm has diminished over time because of early intervention, the sepsis mortality rate of ~20% is still excessive (14, 15). Mortality from sepsis is in part due to the complexity and interconnectedness of the cytokine storm that is composed of both pro- and anti-inflammatory cytokines (16–18) and is further complicated by individual comorbidities (19, 20). The underlying link between MS and subsequent sepsis is not clear. MS patients are often prescribed one of several immunosuppressant drugs, putting them at greater risk of infection. Indeed, certain disease-modifying therapies for MS pose a greater risk for infection, such as rituximab, compared with others (21).

Patients with autoimmune diseases, such as MS, are often treated with immunomodulatory drugs that may increase their susceptibility to infection and sepsis. For example, urinary tract infection and respiratory infection are both common causes of sepsis (22) and complications for MS patients, relative to the general population (23, 24). In fact, compared with the general healthy population, individuals with MS are at greater risk of sepsis, sepsis-induced complications, and death because of infection (25). MS patients are also more likely to have a principal diagnosis of infection at their final hospital stay prior to death compared with the general healthy population and individuals with diabetes mellitus (26). Moreover, sepsis was a secondary diagnosis for 51% of MS patients compared with 36 and 31% of diabetes mellitus and general healthy individuals, respectively, during a hospital stay (26), demonstrating that even among autoimmune disease, MS patients are at increased risk of developing sepsis. The increased propensity to become septic also extends to military veterans, a population that is skewed toward individuals >50 y of age and male (27), both of which are associated with an increased prevalence of sepsis. Last, veterans with MS are more likely to be hospitalized and die of infection compared with veterans without MS (28).

We previously studied the impact of sepsis on subsequent MS-like disease using the experimental autoimmune encephalomyelitis (EAE) animal model as a means of conceptually interrogating the immunoparalysis state that occurs after sepsis (29). However, there is a strong need to understand how underlying autoimmune conditions, such as MS, influence susceptibility to sepsis-induced mortality, given the increased prevalence in this potentially vulnerable population. Thus, with the present study, we affirm the increased prevalence of sepsis in MS patient cohorts relative to non-MS patient cohorts and interrogate how autoimmunity as a comorbidity in septic populations influences susceptibility to sepsis-induced mortality using murine models of MS (EAE) sepsis (cecal ligation and puncture [CLP], LPS, and *Streptococcus pneumoniae*).

## MATERIALS AND METHODS

### Retrospective patient assessment

TriNetX was used to query a limited, deidentified dataset of patients at the University of Iowa admitted between 2008 and 2020. Adult patients (age 18–119 y) who had inpatient encounters were queried. Because this period spans the transition from the International Classification of Diseases (ICD) 9 to ICD-10 coding, the TriNetX software uses algorithms to transform ICD-9 codes to ICD-10 codes. Sepsis patients were queried for all ICD-10 codes including sepsis in their description using the [or] operator. MS patients were queried using ICD-10 code group G35 MS. TriNetX is compliant with the Health Insurance Portability and Accountability Act (HIPAA), the U.S. federal law that protects the privacy and security of health care data. TriNetX is certified to the Information Security Officer 27001:2013 standard and maintains an Information Security Management System to ensure the protection of the health care data it has access to and to meet the requirements of the HIPAA Security Rule. Any data displayed on the TriNetX Platform in aggregate form or any patient level data provided in a dataset generated by the TriNetX Platform only contain deidentified data as per the deidentification standard defined in Section 164.514(a) of the HIPAA Privacy Rule. The process by which the data are deidentified is attested to through a formal determination by a qualified expert as defined in Section 164.514(b) (1) of the HIPAA Privacy Rule.

### Ethics statement

Experimental procedures using mice were approved by University of Iowa Animal Care and Use Committee under Animal Care and Use Review Form protocol no. 6121915 and no. 9101915. The experiments performed followed Office of Laboratory Animal Welfare guidelines and Public Health Service Policy on Humane Care and Use of Laboratory Animals. Cervical dislocation was used as the euthanasia method of all experimental mice.

### Mice

Inbred male and female C57BL/6 (Thy1.2/1.2) mice were purchased from the National Cancer Institute (Frederick, MD) and maintained in the animal facilities at the University of Iowa at the appropriate biosafety level. Genders were equally represented across experimental groups in experiments.

### CLP model of sepsis induction

CLP surgery was performed as previously described (30). Briefly, mice were anesthetized with ketamine/xylazine (University of Iowa, Office of Animal Resources), the abdomen was shaved and disinfected with Betadine (Purdue Products), and a midline incision was made. The distal third of the cecum was ligated with Perma-Hand Silk (Ethicon), punctured once using a 25-gauge needle, and a small amount of fecal matter was extruded. The cecum was returned to abdomen, the peritoneum was closed with 641G Perma-Hand Silk (Ethicon), and skin was sealed using surgical Vetbond (3M). Following surgery, 1 ml PBS was administered s.c. to provide postsurgery fluid resuscitation. Lidocaine was administered at the incision site, and flunixin meglumine (Phoenix Scientific) was administered for postoperative analgesia. This procedure created a septic state characterized by loss of appetite and body weight, ruffled hair, shivering, diarrhea, and/or periorbital exudates, with 0–10% mortality rate. Sham mice underwent identical surgery excluding CLP.

### LPS endotoxemia induction

Mice received a single i.p. injection of LPS from *Escherichia coli* O55:B5 (2.5 mg/kg body weight; Sigma), as previously described (31).

### S. pneumoniae *infection*

*Streptococcus* was grown in brain heart infusion (BHI) broth and then pelleted by centrifugation. Pellet was washed three times and diluted to a target absorbance of 0.1 using PBS, as measured by ABS_600_. Mice were anesthetized with ketamine/xylazine and received 40 μL of *S. pneumoniae* by intranasal inoculation. Infectious dose was confirmed by plating inoculum (1.5 × 10^6^ CFU/mouse) on BHI plates.

CFU per gram of lung was determined by sacrificing mice and weighing the lungs. Lungs were mechanically homogenized in 1 ml of PBS. Twenty microliters of homogenate on BHI plates in duplicate.

### EAE disease induction and evaluation

EAE was induced and evaluated as shown previously (32). Briefly, mice were immunized s.c. on day 0 on the left and right flank with 100 μg of MOG_35–35_ emulsified in CFA followed by 80 ng of pertussis toxin i.p. on days 0 and 2. Disease severity was scored as follows: 0, no clinical symptoms; 1, loss of tail tonicity; 2, hind limb weakness; 3, hind limb paralysis; 4, fore limb weakness; and 5, moribund or death.

### Cytokine analysis

Multiplex cytokine analysis was performed via Thermo Fisher Scientific ProcartaPlex 7-Plex, according to the manufacturer’s instructions for plasma cytokine analysis. Multiplex was analyzed on Bio-Rad Bio-Plex (Luminex 200) analyzer in the University of Iowa Flow Cytometry core facility.

IL-6 and IL-10 ELISAs (ELISA MAX Deluxe Set; BioLegend) were performed according to the manufacturer’s instructions.

### Statistical analysis

Unless stated otherwise, data were analyzed using Prism 8 software (GraphPad Software) using two-tailed Student *t* test (for two individual groups, if variance was unequal, then Mann–Whitney *U* test), one-way ANOVA with Bonferroni post hoc test (for more than two individual groups, if variance was unequal, then Kruskal–Wallis with Dunn post hoc test was used), and two-way ANOVA (for multiparametric analysis of two or more individual groups, pairing was used for samples that came from the same animal) with a confidence interval of >95% to determine significance (*p < 0.05). Log-rank (Mantel–Cox) curve comparisons were used to determine a significant difference in time to EAE disease onset (*p < 0.05). Data are presented as SEM.

## RESULTS

### MS patients are more prone to sepsis than the general population

Prior literature suggests an increased susceptibility of MS patients to develop sepsis relative to non-MS patient cohorts (25). Therefore, to begin interrogating this potentially interesting interplay, we performed a retrospective analysis of intensive care unit admissions at the University of Iowa Hospital and Clinics. This analysis included 211,470 patients admitted between 2008 and 2020, of which there were 22,930 that were septic and 1180 that had MS (Table I). Notable features of these patient cohorts included that septic patients tended to be older and male, known risk factors associated with developing sepsis (19, 20), whereas MS patients tended to be female; MS is a known female-biased disease (1). There was also a slight increase in the proportion of white patients among the septic patients. Importantly, MS patients exhibited a significant increase in sepsis prevalence (14.4%) relative to non-MS patients (10.8%; odds ratio: 1.387, *p* = 0.0001) (Table I). Additionally, whereas MS patients tended to be female, there was a higher proportion of males among the septic MS patients (35%) relative to the nonseptic MS patients (26%) (Table I). Further, septic MS patients also tended to be older (64 ± 14 y) than their nonseptic MS patient counterparts (56 ± 16 y) (Table I). These data reaffirm both the higher prevalence of sepsis in males and with age, even within the MS patient cohort. Overall, these data affirm that MS patients have an increased prevalence of sepsis relative to non-MS patient cohorts.

### EAE increases host susceptibility to sepsis-induced mortality

**FIGURE 1.**
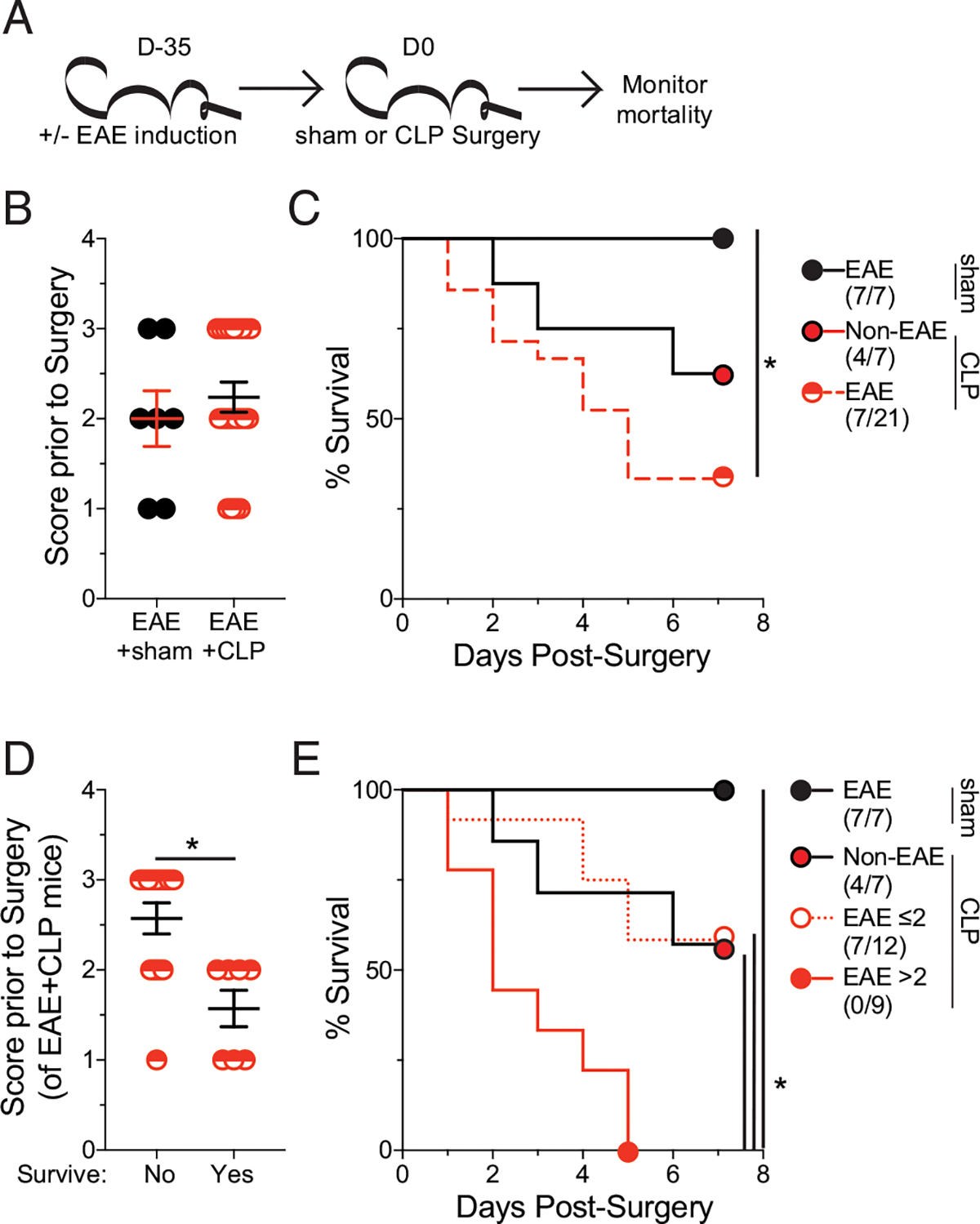
EAE mice have increased susceptibility to sepsis-induced mortality. (**A**) Experimental design: C57BL/6 mice were immunized with MOG_35–35_ to induce EAE. EAE mice underwent either sham or CLP 35 d after EAE induction followed by assessment of mortality age-matched nonimmunized (non-EAE) underwent CLP surgery at the same time. (**B**) EAE clinical scores of mice prior to either sham or CLP surgery. (**C**) Kaplan–Meier survival curves of EAE mice that underwent sham (black closed circle) or CLP (red semicircle) surgery and non-EAE mice that underwent CLP surgery (red closed circle with black outline). (**D**) EAE clinical scores prior to surgery of EAE mice that either succumbed to or survived the septic insult. (E) Kaplan–Meier survival curves of EAE mice that underwent sham (black circle), had an EAE score ≤2 prior to CLP (white circle with red outline), or had an EAE score >2 prior to CLP (red closed circle with red outline) surgery and non-EAE mice that underwent CLP surgery (red closed circle with black outline). Data are cumulative of two independent experiments with 7–21 mice per group. Error bars represent SEM. *p < 0.05.

Given that MS patients have a higher prevalence of sepsis, we sought to understand how having an ongoing autoimmune disease would influence host susceptibility to sepsis. To address this relationship, well-established models of inducible MS-like disease and polymicrobial sepsis, EAE and CLP, respectively, were used. C57BL/6 mice were immunized with MOG_35–35_ to induce EAE or left unimmunized (non-EAE). CLP or sham surgery was performed >35 d postimmunization, and mortality was assessed (Fig. 1A). To ensure that mortality was not simply because of ongoing EAE disease, EAE mice were segregated into sham and CLP groups to establish a similar distribution of EAE clinical scores prior to surgery (Fig. 1B). Non-EAE mice exhibited some mortality; however, EAE mice had diminished survival relative to non-EAE mice (Fig. 1C). Importantly, EAE mice that underwent sham surgery did not have any mortality, consistent with the model system and demonstrating that mortality in EAE with CLP was not because of EAE disease. These data also suggest the presence of CNS autoimmunity increases the host susceptibility to a fatal septic event. Interestingly, there was an observed relationship between the EAE disease score prior to sepsis induction and the likelihood of mortality (Fig. 1D,1E). Mice with a score of ≤2 had a similar survival rate to naive CLP mice, whereas all mice with an EAE score >2 succumbed to disease (Fig. 1E).

### Autoimmune inflammation, not clinical disease, dictates susceptibility to sepsis

**FIGURE 2.**
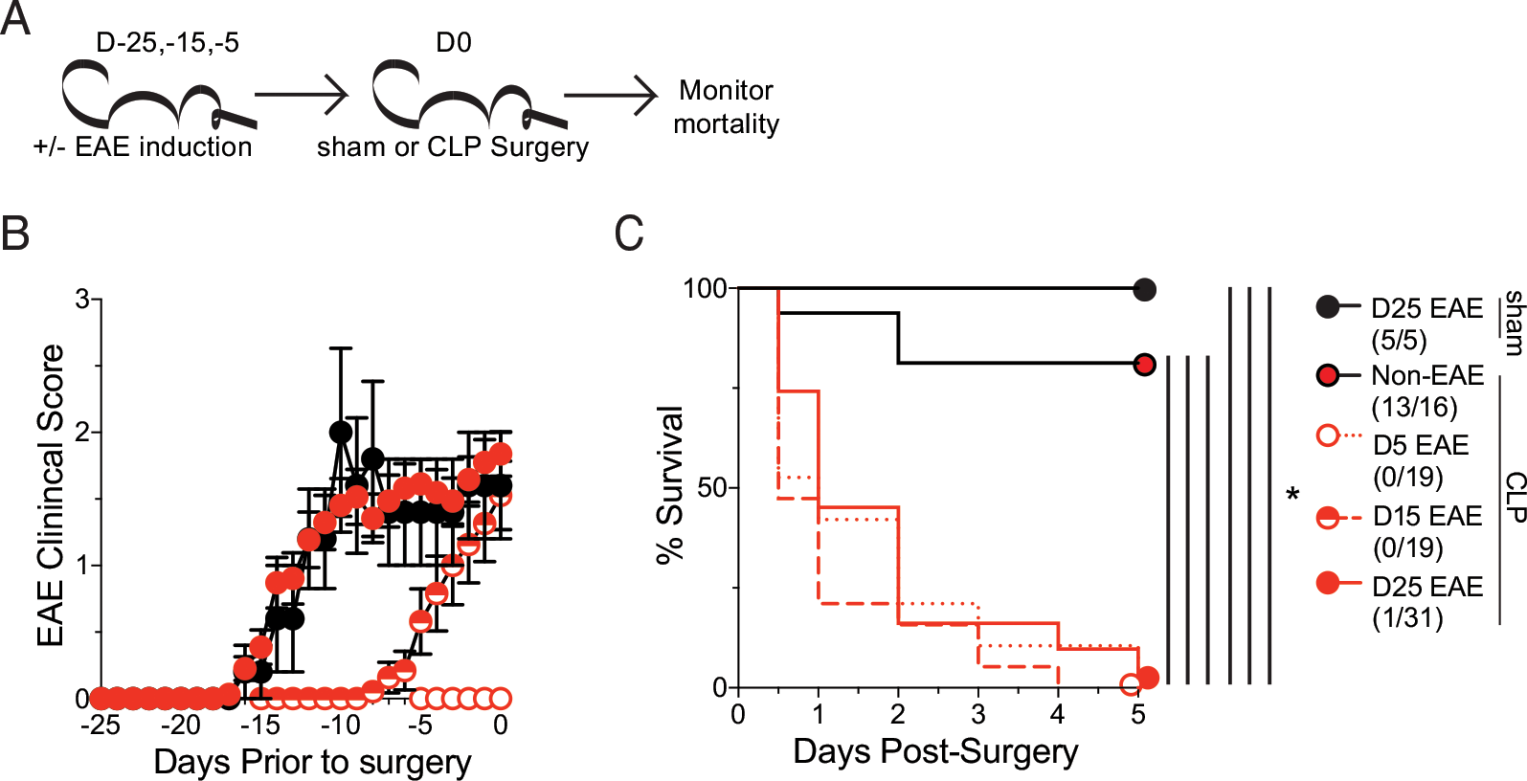
Increased susceptibility of EAE mice to sepsis is independent of disease onset. (**A**) Experimental design: C57BL/6 mice were immunized with MOG_35–35_ to induce EAE at day −25, −15, or −5 prior to either sham or CLP surgery, and age-matched nonimmunized (non-EAE) underwent CLP surgery at the same time. Mortality was monitored in all cohorts. (**B**) EAE clinical scores of mice that were induced for EAE at −25, −15, −5 prior to either sham or sepsis surgery. (**C**) Kaplan–Meier survival curves of day −25 EAE mice that underwent sham surgery (black circle), non-EAE mice that underwent sepsis surgery (red circle with black outline), and day −25 (red circle with red outline), day −15 (red semicircle), and day −5 (white circle with red outline) EAE mice that underwent CLP. Data are cumulative of two independent experiments with 5–31 mice per group. Error bars represent SEM. *p < 0.05.

The relationship between disease severity and mortality suggests that either the paralysis and associated neurologic damage during EAE is promoting sepsis-induced mortality, or differences in the inflammatory response may increase the likelihood of mortality. Indeed, we previously reported that microbially experienced “dirty” mice with a high degree of immunologic experience are highly susceptible to sepsis-induced mortality due (in part) to elevations in plasma cytokine concentrations both at a baseline and during the peak (~12 h postinduction) of the cytokine storm (31). Similarly, we have also described a relationship between tumor size at the time of sepsis induction and host mortality (12). Thus, to begin teasing apart the roles of the interconnected phenomena of inflammation and paralysis, mice were immunized at varying times leading up to sepsis induction. This approach establishes a scenario in which disease is subclinical (day 5 [D5]), being established (day 15 [D15]), or fulminant (day 25 [D25]), with ongoing inflammation anticipated in all cohorts (Fig. 2A). Clinical disease progression occurred in agreement with these expectations (Fig. 2B). All EAE cohorts, however, exhibited profound susceptibility to sepsis-induced mortality, demonstrating that clinical disease and paralysis were not required for sepsis-induced mortality (Fig. 2C).

**FIGURE 3.**
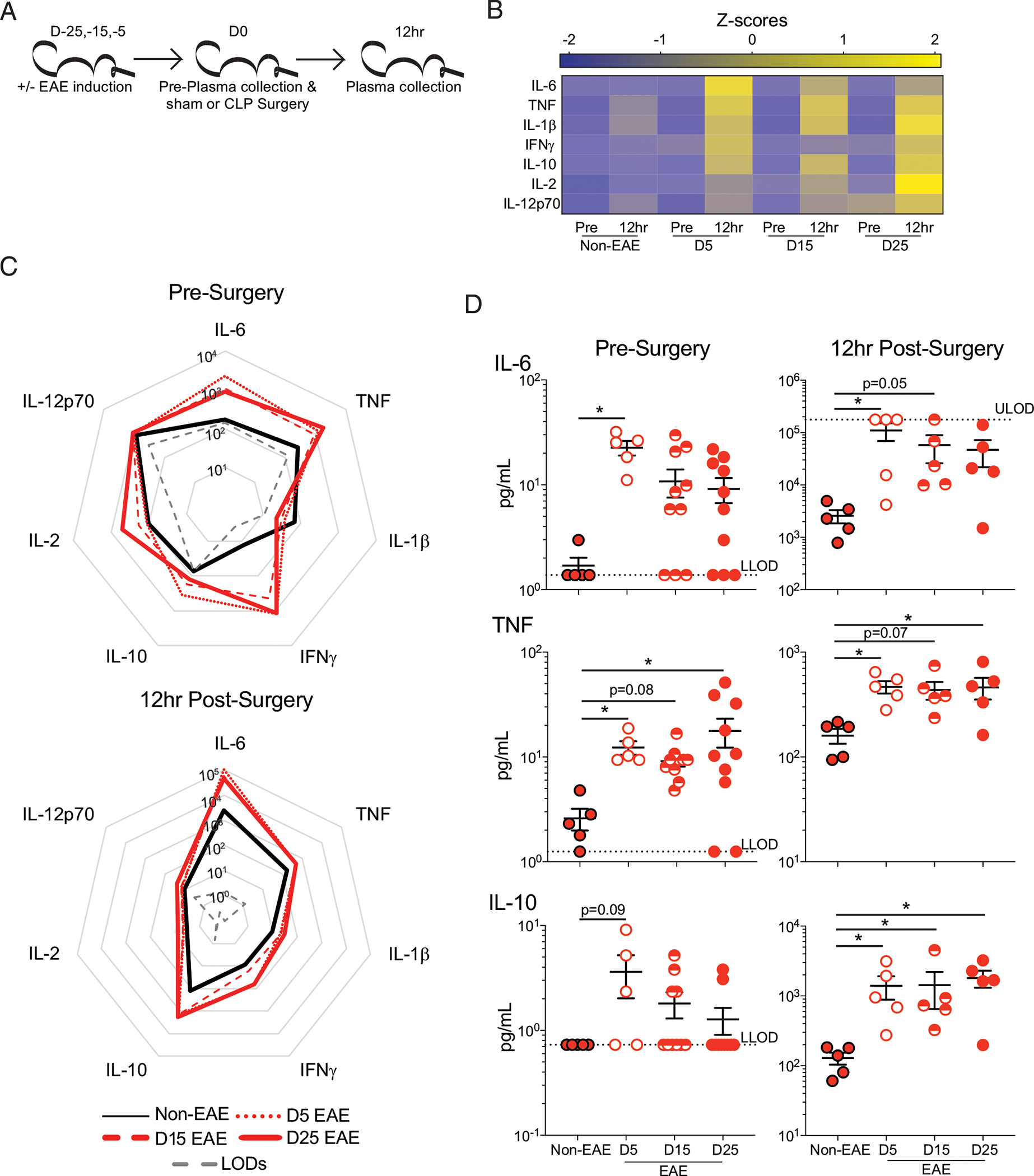
EAE mice have increased inflammation prior to and following sepsis induction. (**A**) Experimental design: C57BL/6 mice were immunized with MOG_35–55_ to induce EAE at day −25, −15, or −5 prior to CLP surgery, and age-matched nonimmunized (non-EAE) underwent CLP surgery at the same time. Plasma was collected prior to surgery and 12 h postsurgery. (**B**) Heatmap of normalized plasma IL-6, TNF, IL-1β, IFN-γ, IL-10, IL-2, and IL-12p70 concentrations in non-EAE, D5EAE, D15 EAE, and D25 EAE mice prior to and 12 h post–CLP surgery. (**C**) Radar plots of plasma IL-6, TNF, IL-1β, IFN-γ, IL-10, IL-2, and IL-12p70 in non-EAE mice (black line), D5 (dotted red line), D15 (dashed red line), and D25 EAE mice (solid red line) prior to (top) and 12 h post– (bottom) CLP surgery. (**D**) Representative plasma cytokines (top to bottom: IL-6, TNF, and IL-10) prior to (left) and 12 h post– (right) CLP surgery in non-EAE (red circle with black outline), D5 EAE (white circle with red outline), D15 EAE (red semicircle), and D25 EAE (red circle with red outline) mice. Gray dashed lines indicate the upper limit of detection (ULOD) and lower limit of detection (LLOD) for the multiplex assay. Samples are combined from two independent experiments run on a single multiplex assay with 5–10 mice per group. Error bars represent SEM. *p < 0.05.

To then address the extent to which EAE, similar to infection and cancer, was altering the severity of the sepsis-induced cytokine storm, plasma was collected prior to and 12 h post–CLP surgery in D5, D15, and D25 (as well as non-EAE) mice and assessed for IL-6, TNF, IL-1β, IFN-γ, IL-10, IL-2, and IL-12p70 (Fig. 3A). Importantly, although there was a cytokine storm in all CLP cohorts, the magnitude of the cytokine storm was substantially higher in EAE mice relative to the non-EAE mice (Fig. 3B–D). Further, EAE mice had a higher baseline expression of many cytokines (Fig. 3C, 3D), recapitulating observations in dirty mice (31). Of particular note was IL-6, which has previously been described as a strong indicator of the severity of the cytokine storm (33–35) and was strongly increased in all EAE groups both prior to and after CLP (Fig. 3D).

**FIGURE 4.**
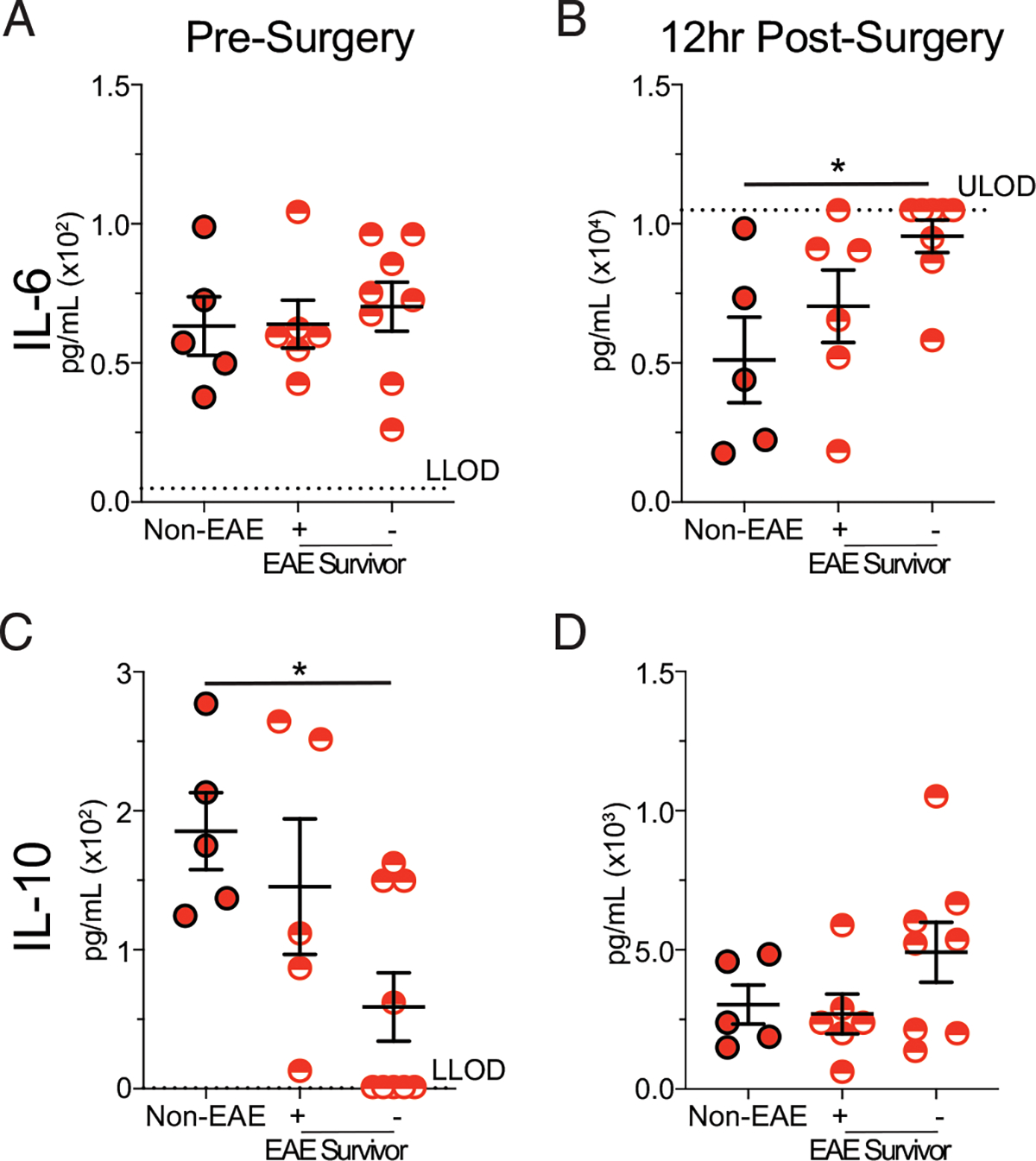
Mortality in EAE mice is associated with elevated inflammation. C57BL/6 mice were immunized with MOG_35–55_ to induce EAE. EAE and age-matched nonimmunized (non-EAE) mice under went CLP 35 d after EAE induction. Plasma cytokines were assessed prior to and 12 h post–CLP surgery in non-EAE, EAE mice that survived CLP-induced sepsis, and EAE mice that succumbed to CLP-induced sepsis. Plasma IL-6 (A and B) and IL-10 (C and D) prior to (A) and (C) and 12 h post– (B) and (D) CLP surgery in non-EAE, EAE mice that survived CLP-induced sepsis, and EAE mice that succumbed to CLP-induced sepsis. Gray dashed lines indicate the upper limit of detection (ULOD) and lower limit of detection (LLOD) for the respective ELISA plate. Samples are combined from two independent experiments run on single ELISA plates with five to eight mice per group. Error bars represent SEM. *p < 0.05.

These results then led us to question whether there was a quantitative difference in the magnitude of the cytokine storm between survivor and nonsurvivor mice at day 35 post–EAE induction. Thus, plasma IL-6 and IL-10 were interrogated in survivor and nonsurvivor EAE mice as well as non-EAE mice prior to and 12 h after EAE induction (Fig. 4). Indeed, nonsurvivor mice had an elevated cytokine storm, whereas survivor mice had a similar magnitude of the cytokine storm as non-EAE mice. This finding further illustrates that the susceptibility of EAE mice to sepsis-induced mortality is through enhancement of the cytokine storm.

### EAE mice have increased susceptibility to various models of sepsis induction

**FIGURE 5.**
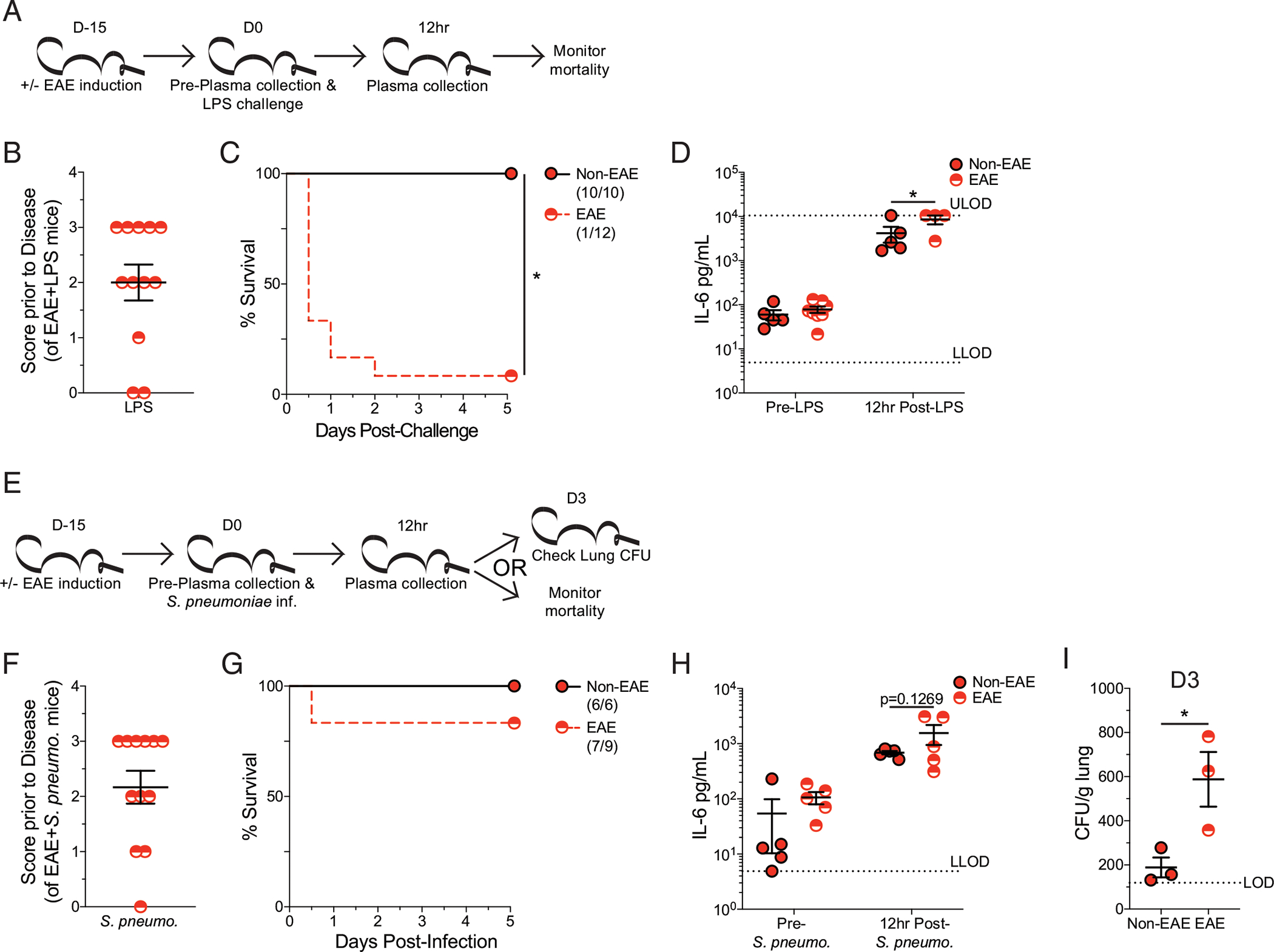
EAE mice have an increased susceptibility to multiple sepsis models. (**A**) Experimental design: C57BL/6 mice were immunized with MOG_35–55_ to induce EAE 15 d prior to i.p. LPS injection, and age-matched nonimmunized (non-EAE) received identical injections. Plasma was collected prior to and 12 h post–LPS injection. Cohorts were monitored for survival. (**B**) EAE disease scores prior to LPS injection of EAE mice. (**C**) Kaplan–Meier survival curves for non-EAE and D15 EAE mice following LPS injection. (**D**) Plasma IL-6 prior to and 12 h post–LPS injection in non-EAE (red circle with black outline) and D15 EAE (red semicircle). Gray dashed lines indicate the upper limit of detection (ULOD) and lower limit of detection (LLOD) for IL-6 ELISA. Data are from a single experiment with 10–12 mice per group. Error bars represent SEM. *p < 0.05. (**E**) Experimental design: C57BL/6 mice were immunized with MOG_35–55_ to induce EAE 15 d prior to intranasal *S. pneumoniae* infection, and age-matched nonimmunized (non-EAE) received identical infections. Plasma was collected prior to and 12 hpost–LPS injection. Three mice from each cohort were used for determining lung CFU at 3 d postinfection. The remaining mice in each cohort were monitored for survival. (**F**) EAE disease scores prior to *S. pneumoniae* infection of EAE mice. (**G**) Kaplan–Meier survival curves for non-EAE and D15 EAE mice following *S. pneumoniae* infection. (**H**) Plasma IL-6 prior to and 12 h post–*S. pneumoniae* infection in non-EAE (red circle with black outline) and D15 EAE (red semicircle). Gray dashed line indicates the lower limits of detection (LLODs) for IL-6 ELISA. (**I**) *S. pneumoniae* CFU per gram of lung tissue 3 d after intranasal infection of non-EAE and D15 EAE mice. Dashed line indicates the limit of detection (LOD). Data are from a single experiment with 9–12 mice per group. Error bars represent SEM. *p < 0.05.

Given the high susceptibility of EAE mice to fatal CLP-induced sepsis, we sought to extend the applicability of this effect to other models of sepsis induction. i.p. injection of LPS is a well-established model of endotoxemia and sepsis with a highly tunable degree of mortality by modulating the concentration of LPS (18, 36). With this system, a dose of LPS that elicits a robust cytokine storm but does not elicit mortality in unmanipulated (e.g., non-EAE) mice was interrogated (31). LPS was injected 15 d post–EAE induction on EAE and non-EAE cohorts, and mortality was monitored throughout with plasma IL-6 evaluated prior to and 12 h post–LPS injection (Fig. 5A). Consistent with prior experiments, EAE mice had a range of disease scores (Fig. 5B). Importantly, whereas non-EAE mice exhibited no mortality, as anticipated, EAE mice exhibited rapid and profound mortality, recapitulating the observations with CLP (Fig. 5C). The enhanced mortality of EAE mice was attributable to increased IL-6 following LPS injection (Fig. 5D), similar to observations with CLP mice. These data demonstrate increased sensitivity to TLR4 stimulation, which likely contributes to the enhanced mortality among EAE mice.

Next, we examined the impact of having EAE followed by an intranasal *S. pneumoniae* infection. *S. pneumoniae* is the most prevalent causative pathogen of community-acquired pneumonia, and *S. pneumoniae* models of sepsis have high clinical relevance as nearly half of all sepsis cases result from this bacterial infection (37). Similar to the LPS endotoxemia model, *S. pneumoniae* infection in this system does not lead to mortality in unmanipulated mice. It does, however, represent a relevant respiratory infection (38), which, along with *S. pneumoniae* infection, are both a common cause of sepsis (22) and a frequent complication among MS patients (24). Further host ability to control the infection can be assessed by determining the number of CFUs in the lungs and plasma cytokines to give an indication of the host ability to mount an inflammatory response and clear infection. Using this system, EAE mice and non-EAE controls were intranasally inoculated with *S. pneumoniae* 15 d post–EAE induction. Plasma IL-6 was evaluated prior to and 12 h post–*S. pneumoniae* infection. Additionally, lung *S. pneumoniae* CFUs were evaluated in three mice from each cohort 3 d postinfection while mortality was monitored in the remaining mice (Fig. 5E). As before, EAE mice exhibited a range of disease severity prior to infection (Fig. 5F) and some mortality subsequent to the insult (Fig. 5G), although this mortality was not significantly different from non-EAE control mice. Further, a trending increase in plasma IL-6 was observed from EAE mice 12 h post–*S. pneumoniae* infection (Fig. 5H), in agreement with the prior findings of an elevated inflammatory response in EAE mice challenged with either CLP or LPS. However, the elevation in IL-6 was not to the same degree as it was seen in the other sepsis models. This potentially reveals a relevant distinction in how autoimmunity (EAE) enhances host susceptibility to sepsis-induced mortality. Some of these relevant factors may include the site of inoculation, stimulus itself, cells activated in response to challenge, and capacity of the insult to evoke a systemic response. Interestingly, EAE mice also had reduced control of *S. pneumoniae* infection 3 d postinfection, relative to non-EAE mice (Fig. 5I). These data indicate that despite enhanced inflammation, EAE mice have a dysregulated inflammatory response that has a reduced capacity to provide protection to subsequent insult. Thus, the culmination of enhanced inflammatory responses with a reduced capacity to control pathogen insult may set the stage for the enhanced susceptibility of EAE mice and MS patients to develop and succumb to septic insults.

## DISCUSSION

Cumulatively, these findings indicate that MS patients are at a higher risk of developing sepsis, and ongoing autoimmune reactions lay the groundwork for an exacerbated inflammatory response during septic insult that in turn increases the risk of mortality. This conclusion is relevant to both the identification and management of patient populations that are likely to become septic and at high risk of mortality in the event they become septic. Future work should interrogate the utility of intervention strategies in promoting the survival of sepsis, and assessments of intervention strategies should account for these highly relevant comorbidities in determining efficacy. Importantly, patients with autoimmunity tend to be on immunosuppressive regimens (5, 6); although it is yet unclear what the net result of these interventions are on the development of sepsis, these immunosuppressive regimens will undoubtedly be pertinent to the management of the cytokine storm. The patient data used in this study is deidentified and lacks the granularity necessary to assess what medications MS patients may be on at the time of septic insult. Future interrogation should be performed to parse the influences of different immunosuppressive regimens on MS patient susceptibility to septic insult. This can be further extended by exploring the interactions between therapies for autoimmunity and interventions during sepsis in mouse models to potentially facilitate patient-specific intervention strategies during sepsis, dependent on the therapy they receive for their autoimmunity.

In addition, it is relevant to consider the classification of MS and its influence on host survival. As with the immunosuppressive medications, our dataset does not provide enough information to address the susceptibility of different MS patient cohorts to MS (e.g., relapsing remitting versus primary progressive). In the current study, we use an EAE model that mimics primary progressive MS. However, relapsing remitting MS is the most common type of MS, and future studies using the SJL model of relapsing remitting MS should be performed. It is interesting to consider that in the current study, clinical disease was not required to enhance survival (i.e., D5 postimmunization). Alternately, EAE mice continued to exhibit enhanced susceptibility to sepsis after the peak inflammatory response has passed (i.e., day 35 postimmunization). This potentially emulates an extended remission, further suggesting that even during remissions, patients may still have an increased susceptibility to sepsis.

Alternately, it is also relevant to consider the consequences for a patient with autoimmunity who survives a septic insult. This notion is highly related to our previous findings, wherein we observed sepsis-induced immunoparalysis ablated the subsequent development of EAE through the numeric reduction in naive autoantigen-specific CD4 T cells (29). Indeed, sepsis similarly reduces the number and function effector and memory T cells (39–43). Therefore, it is plausible for those individuals that survive to experience a reduction in their autoimmune disease symptoms. Contrastingly, sepsis may also reduce the capacity of suppressor cell populations to mediate their activity and lead to disease exacerbation (44–46). There are likely multiple complicating factors that dictate whether any such benefit or detriment arises, including the stage of autoimmune disease progression. Such interrogation may lead to enhanced understanding of the sepsis-induced immunoparalysis state or even future therapeutic intervention for MS and autoimmune disease patients.

Finally, it is relevant to consider the observation that clinical disease was not required for the enhancement in mortality among EAE mice. This finding suggests individuals with subclinical or newly developing autoimmunity may be at risk for increased mortality from sepsis. This possibility may be problematic for delineating patient populations with high susceptibility to sepsis-induced mortality as it may not be a recognized complicating factor. Thus, enhanced susceptibility of patient populations to sepsis-induced mortality may be better understood as a result of active inflammatory responses prior to septic insult rather than highly specific comorbidities such as autoimmunity or cancer. These are highly relevant notions when seeking to promote survival and develop future therapeutics.

## Figures and Tables

**TABLE I.** Prevalence of sepsis among MS and non-MS patients at University of Iowa Hospitals and Clinics

Patient Cohort	All Inpatient	Nonseptic	Septic	Septic (%)	Nonseptic versus Septic	MS versus Non-MS

Total	211,470	188540	22,930	10.8		
Age (±SD)	58 ± 20	57 ± 21	64 ± 17		<0.0001	
Male (%)	47	46	53		<0.0001	
White (%)	87	86	88		<0.0001	
Non-MS	210,290	187,530	22,760	10.8		
Age (±SD)	60.5 ± 5	57 ± 21	64 ± 17		<0.0001	
Male (%)	47	46	53		<0.0001	
White (%)	86	86	89		<0.0001	
MS	1,180	1,010	170	14.4		
Age (±SD)	60 ± 6	56 ± 16	64 ± 14		<0.0001	NS
Male (%)	27	26	35		0.0159	<0.0001
White (%)	89	89	88		NS	0.0121
Sepsis odds ratio	1.387					0.0001

## Abbreviations used in this article:

BHI: brain heart infusion
CLP: cecal ligation and puncture
D5: day 5
D15: day 15
D25: day 25
EAE: experimental autoimmune encephalomyelitis
HIPAA: Health Insurance Portability and Accountability Act
ICD: International Classification of Diseases
MS: multiple sclerosis
